# Blood-brain barrier hyperpermeability precedes demyelination in the cuprizone model

**DOI:** 10.1186/s40478-017-0497-6

**Published:** 2017-12-01

**Authors:** Stefan A. Berghoff, Tim Düking, Lena Spieth, Jan Winchenbach, Sina K. Stumpf, Nina Gerndt, Kathrin Kusch, Torben Ruhwedel, Wiebke Möbius, Gesine Saher

**Affiliations:** 1 Department of Neurogenetics, Max-Planck-Institute of Experimental Medicine, Hermann-Rein-Str. 3, 37075 Goettingen, Germany; 2 Center Nanoscale Microscopy and Molecular Physiology of the Brain (CNMPB), Wilhelmsplatz 1, 37073 Göttingen, Germany

**Keywords:** Blood-brain barrier, Demyelination, Gliosis, Astrocyte, Inflammatory mediators

## Abstract

In neuroinflammatory disorders such as multiple sclerosis, the physiological function of the blood-brain barrier (BBB) is perturbed, particularly in demyelinating lesions and supposedly secondary to acute demyelinating pathology. Using the toxic non-inflammatory cuprizone model of demyelination, we demonstrate, however, that the onset of persistent BBB impairment precedes demyelination. In addition to a direct effect of cuprizone on endothelial cells, a plethora of inflammatory mediators, which are mainly of astroglial origin during the initial disease phase, likely contribute to the destabilization of endothelial barrier function in vivo. Our study reveals that, at different time points of pathology and in different CNS regions, the level of gliosis correlates with the extent of BBB hyperpermeability and edema. Furthermore, in mutant mice with abolished type 3 CXC chemokine receptor (CXCR3) signaling, inflammatory responses are dampened and BBB dysfunction ameliorated. Together, these data have implications for understanding the role of BBB permeability in the pathogenesis of demyelinating disease.

**Electronic supplementary material:**

The online version of this article (10.1186/s40478-017-0497-6) contains supplementary material, which is available to authorized users.

## Introduction

The cerebral vasculature controls and restricts the transport of biomolecules between blood and the CNS parenchyma by means of the blood-brain barrier (BBB) [1, 37]. While specialized brain endothelial cells are physically connected via unique belt-like tight junctions that mediate BBB tightness, perivascular cells and astrocytes also contribute to BBB physiology, collectively forming the neurovascular unit (NVU). In a wide range of neurological disorders including multiple sclerosis (MS) increased vascular permeability has been observed [33] but the primary cause for the pathophysiology of the NVU and the relation to disease specific pathomechanisms remains unclear.

MS is an acquired inflammatory demyelinating disease of the CNS in which BBB permeability is increased in both newly forming demyelinating lesions and even in normal appearing white matter [18, 59]. BBB impairment is observed at the onset of clinical symptoms in experimental autoimmune encephalomyelitis (EAE), an animal model of MS, coinciding with initial immune cell infiltration and glial activation [19, 51]. In this model, pro-inflammatory cytokines/chemokines produced by activated immune cells on the peripheral side of the barrier, or by glial cells in the CNS, contribute to BBB hyperpermeability [4, 42]. Thus, BBB disruption could potentially be secondary to pathology. However, it is unclear whether demyelination or other disease factors cause BBB disturbances.

We previously showed in the non-inflammatory cuprizone model of demyelination [45], that BBB permeability is increased at the peak of demyelinating disease, and that this BBB dysfunction can be utilized for CNS delivery of therapeutics [11]. However, it is unclear which pathomechanism triggers the BBB breach in the cuprizone model. Here, we further characterize these BBB disturbances, relating BBB pathophysiology to histopathology in different brain regions and at different stages of disease progression. We demonstrate that early disease processes are associated with elevated levels of several pro-inflammatory mediators of predominantly astroglial origin. This local inflammatory milieu, together with a primary effect of cuprizone on endothelial cells, leads to the downregulation of BBB maintenance factors, endothelial efflux transporters, and tight junction proteins resulting in morphological disruption of tight junctions. These endothelial disturbances are associated with local hyperpermeability of the BBB and edema, even before the onset of demyelination.

## Materials and methods

### Mice

All animal studies were performed in compliance with the animal policies of the Max Planck Institute of Experimental Medicine, and were approved by the German Federal State of Lower Saxony. Adult male C57BL/6N mice or CX3CR1^GFP/GFP^ mice [26] were taken at 8–10 weeks of age. Animals were randomly assigned to an experimental group. Cuprizone (0.2% *w*/w, Sigma) was mixed into powder chow (V1124 ssniff).

### Histological analyses

Histological analyses were done as described [11, 49] with minor modifications. Anesthetized mice were perfused with paraformaldehyde (PFA) and brains were cut by vibratome (40 μm, Leica VT1200)) or embedded in paraffin. Brain sections (HMP 110, MICROM) at Bregma −1.58 were taken for histological characterization using standard protocols using LSAB2 (Dako) or Vector Elite ABC (Vector Labs). For occludin and ZO1 staining, animals were perfused with PFA containing 0.2% glutaraldehyde and cut on a vibratome. Tissue sections or fixed endothelial cells were processed for immunolabeling by permeabilization (0.4% Triton X-100 in PBS), blocking (4% horse serum, 2% BSA, 0.2% Triton X-100 in PBS) and incubation with first antibody (1% HS, 0.05% Triton X-100 in PBS). Incubation with fluorophore coupled secondary antibodies (ThermoFisher) and DAPI (4′,6-diamidino-2 phenylindole) were done in 1.5% HS in PBS, after which sections were mounted in AquaPolymount (Polysciences). Gallyas silver impregnation was done as described [49]. Specimens were analyzed on an Axio Imager.Z1 (Zeiss) equipped with an AxioCam MRc3, × 0.63 Camera Adaptor and the ZEN 2012 blue edition software using ×10 objective (Plan Apochromat × 10/0.45 M27) or × 20 objective (Plan-Apochromat × 20/0.8) or by confocal laser scanning microscopy (Leica SP5 HCX PL APO CS 63×/1.20) using the Leica Confocal Software (Leica Microsystems). Quantification of positive areas in the corpus callosum above the fornix were done by semi-automated analysis with ImageJ software macro and color deconvolution plug-in. Vessel paint was performed as described [34] with minor modifications. Mice were intravenously injected with 200 μl of 20 mg/ml FITC-Dextran (46,945, Sigma-Aldrich Inc., Germany). After 30 min circulation time animals were anaesthetized, flushed, stained with DID (ThermoFischer, D7757) and fixed with PFA before sectioning with a vibratome (100 μm). All Images were processed with NIH ImageJ and Adobe Photoshop CS5.1 software. Electron microscopic analysis was done as previously described [49]. Briefly, tissue was fixed in 4% PFA, 2.5% glutaraldehyde, 0.1 M Phosphate buffer and sagittal sections were cut on a vibratome (Leica VT1200). The corpus callosum with adjacent tissue was punched and embedded in epon (LYNXII, EMS). Ultrathin uranyl acetate contrasted sections were imaged with a LEO EM912 AB (Zeiss) equipped with a 2 k–CCD camera (TRS, Moorenweis).

### Blood-brain barrier permeability

Tracer injections were done as described [11, 49] with minor modifications. For measurement of BBB permeability, tracers were i.v. injected (Evans blue 50 mg g^-1^ body weight; sodium fluorescein 200 mg g^-1^ body weight). After 4 h incubation, animals were perfused with PBS to remove tracer from the circulatory system. The region of interest was dissected, and tissue samples were weighed. For lyophilization, tissues were exposed to a shelf temperature of −56 °C for 24 h under vacuum of 0.2 mBar (Christ LMC-1 BETA 1–16). Samples were weighed for calculation of water content and edema. Lyophilized tissue samples were extracted with formamide at 57 °C for 24 h on a shaker at 300 rpm (Eppendorf Thermomixer). Integrated density of tracer fluorescence was determined in triplicates after 1:3 ethanol dilution to increase sensitivity. Tracer concentration was calculated using a standard curve of tracer spiked brain samples.

### Immunoblot

Brain tissue samples were lysed in sucrose buffer (18% sucrose, 10 mM Tris/HCl pH 7.4, 1 mM sodium hydrogen carbonate, 1 mM magnesium chloride, 0.1% Triton, 0.2% lithiumdodecyl sulphate, 0.025% sodium deoxycholate) with protease inhibition (Roche) using a Precellys 24 homogenizer (Bertin technologies). Detection of immunolabeled proteins was performed with ECL detection reagent (Perkin Elmer) using ChemoCam Imager (Intas).

### Magnetic cell isolation

Glial cells were isolated according to the adult brain dissociation protocol (Miltenyi biotec). Corpus callosum and cortex were isolated using a brain matrix from Bregma +1.10 to −2.46. Antibody labeling steps were done according to the respective antibody Microbead kit protocol (Miltenyi biotec), oligodendrocytes (O4, 130–096-670); astrocytes (ACSA-2, 130–097-679), microglia (CD11b, 130–093-636), and endothelial cells (CD31, 130–091-935). Purity of cell populations was routinely determined by qPCR on extracted and reverse transcribed RNA (see below) and revealed only minimal contamination by other cell types.

### Cell cultures

Primary mouse brain endothelial cell cultures were established from 7 days old mice or rats. Briefly, cortices were digested with 1 mg/ml collagenase/dispase and 2.5 μg/ml DNAse (Roche) in dissection buffer (HBSS, 10 mM HEPES, 0.5% BSA, 5000 U/ml penicillin/streptomycin) for 45 min at 37 °C. After trituration, cells were resuspended in 25% BSA and centrifuged at 1000 g for 20 min to pellet microvessels. Isolated microvessels from individual mice were plated in Endobasal Medium (Promocell) with 0.4% puromycin for positive selection on coverslips or polyester transwell inserts (Corning). Primary astrocyte cultures were prepared from 0 to 2 days old mice as previously described [11]. Primary microglia cultures were prepared from P0-P2 old mice by differential shaking of mixed glial cultures as described [55]. Cell purity was routinely determined by immune stainings and always exceeded 95%. For coculture experiments, endothelial cells cultured in transwell inserts above astrocytes plated on the bottom of the well plate. Confluent cells cultures were treated with a final concentration of 250 μM cuprizone in 0.125% DMSO or in 0.125% DMSO alone for up to 72 h. An epithelial Voltohmmeter (EVOM2, World Precision Instruments) equipped with Endohm-12 chamber electrodes was used to measure transendothelial electrical resistance (TEER). Metabolic activity was determined using a WST1 assay (Cayman) according to the manufactures protocol, after exposure to increasing concentrations of cuprizone (0–250 μM) for up to 72 h, or 20 μM peroxide as positive control.

### Expression analyses

Expression analyses were done as described [11]. For tissue expression analyses, corpus callosum and cortex was dissected from Bregma +1.10 to −2.46. RNA was extracted using QIAshredder and RNeasy protocols (Qiagen). Concentration and quality of RNA was evaluated using a NanoDrop spectrophotometer and RNA Nano (Agilent). cDNA was synthesized with Superscript III (Invitrogen) and quantitative PCRs were done in triplicates with the GoTaq pPCR Master Mix (Promega) on a 7500 Fast Real-Time PCR System (Applied Biosystems). Expression values were normalized to the mean of two housekeeping genes, HPRT (Hypoxanthin-Phosphoribosyl-Transferase 1) and Rplp0 (60S acidic ribosomal protein P), and quantification was done by applying the ΔΔCt method, normalized to age matched untreated controls (set to 1). All primers (Additional file 1: Table S1) were intron-spanning.

### Antibodies

CAII (Said Ghandour), Olig2 (Charles Stiles/ John Alberta), GFAP (Chemicon), MAC3 (Pharmigen), Iba1 (Wako), PECAM1 (dianova), AQP4 (Santa Cruz), occludin, ZO-1 and claudin-5 (Thermo Fisher), GAPDH (Enzo). For generation of GLUT1 antisera, rabbits were immunized with the C-terminal intracellular peptide (CDKTPEELFHPLGADSQV). Anti-GLUT1 antibody was purified by affinity chromatography.

### Statistical analysis

Statistical analysis was performed using Prism software (GraphPad Software), and results are presented as the mean ± s.e.m.. Two-way ANOVA, one-way ANOVA and two-tailed unpaired Student’s t tests were performed as appropriate. Only *P* values <0.05 were considered statistically significant (*P < 0.05, **P < 0.01, ***P < 0.001).

## Results

### Vascular permeability is increased at the peak of cuprizone induced demyelination

**Fig. 1:**
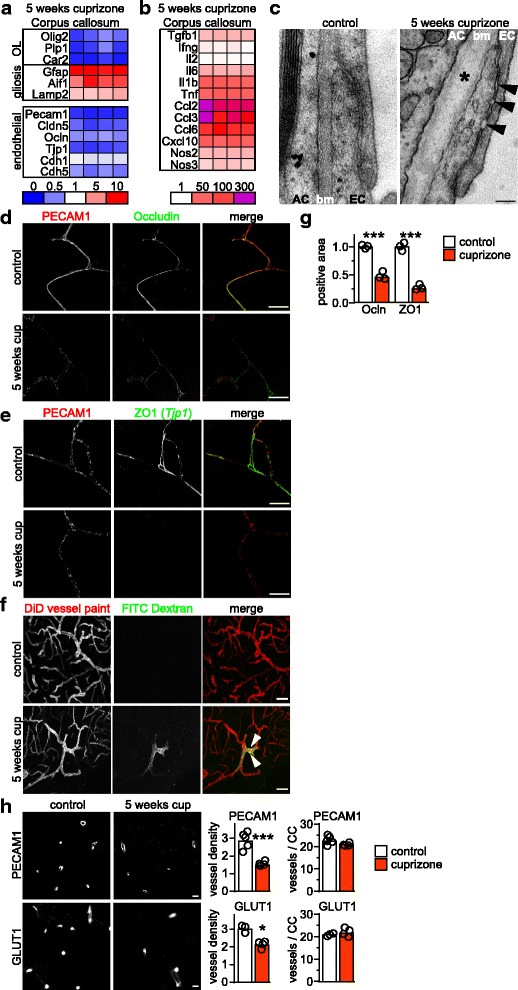
Increased BBB permeability at 5 weeks cuprizone is mediated by downregulation of tight junction proteins. **a**, **b** Quantitative RT-PCR analysis on dissected corpus callosum tissue samples from mice after 5 weeks cuprizone administration. Results show fold-change values of individual animals (*N* = 4) normalized to the mean of untreated control animals (set to 1; *N* = 4–8) according to color code. **a** Assessed were oligodendroglial genes (OL), astroglial and microglial markers (gliosis) and the endothelial markers. **b** Assessed were the inflammatory markers as indicated. **c** Representative electron micrographs of the neurovascular junction in control and cuprizone exposed animals. Arrowheads mark the separation between tight junctions of adjacent endothelial cells, and the asterisk points to split basement membranes (bm) (AC, astrocyte, EC, endothelial cell, Scale bar: 100 nm) (**d**-**e**) Representative pictures of the corpus callosum of untreated control mice and mice after 5 weeks cuprizone exposure immunostained for endothelial proteins as indicated (Scale bars: 20 μm). **f** Extravasation of the tracer FITC-dextran (70 kDa) in mice fed cuprizone for 5 weeks (arrowheads) but not in control mice. DiD vessel paint mark vessel outline (Scale bars: 50 μm). **g** Quantification of abundance of the tight junction proteins occludin (Ocln) and ZO1 from pictures as in (**d**) and (**e**) (*N* = 3 animals per group, Student’s t-test, *P* < 0.001). **h** Density of blood vessels (GLUT1 or PECAM1 positive vessels per 10,000 square μm) in the corpus callosum of control mice (*N* = 5) and mice after 5 weeks cuprizone (*N* = 4). Significance to control was evaluated by Student’s t-test (***P < 0.001). Total number of vessels per corpus callosum (vessels / CC) as evaluated by PECAM1 or GLUT1 staining is similar in both treatment groups (right panel)

At the peak of demyelination after 5 weeks of cuprizone exposure, vascular permeability is increased as we showed previously [11]. To explore potential causes for this BBB instability, we performed a series of expression analyses on dissected corpus callosum samples from mice fed cuprizone for 5 weeks. As expected, histopathology (Additional file 2: Figure S1a) was reflected in reduced expression of oligodendroglial genes, e.g. *Olig2 (*oligodendrocyte lineage transcription factor 2) and increased expression of markers for gliosis (*Gfap,* glial fibrillary acidic protein; *Aif1,* allograft inflammatory factor 1, encoding Iba1) (Fig. 1a, Additional file 1: Table S2). When we quantified the levels of inflammatory mediators that have previously been shown to induce BBB dysfunction [42, 47, 58], *Tnf* (tumor necrosis factor), *Il1b* (interleukin 1 beta), and *Ccl2* (C-C Motif Chemokine Ligand 2) were strongly upregulated (Fig. 1b). Other factors such as *Il2* (interleukin 2) and *Ifng* (interferon gamma) remained unchanged compared to controls. Notably, BBB impairment in the cuprizone model was not associated with mRNA upregulation of HIF1α (hypoxia induced factor 1 alpha), VEGF-A (vascular endothelial growth factor A) or matrix metalloproteinases, which are jointly associated with hypoxia-induced BBB dysfunction in EAE and other disease models [5, 6] (not shown). In contrast, mRNA abundance of the nitric oxide synthases NOS2 and NOS3 was significantly increased in cuprizone treated animals, implicating reactive nitrogen species likely mediators of BBB leakage. Consistent with these findings, expression of endothelial markers including genes encoding tight and adherens junction proteins PECAM1 (platelet/endothelial cell adhesion molecule 1/CD31, *Pecam1* gene), claudin-5 (*Cldn5*), occludin (*Ocln*), ZO1 (*Tjp1* gene), cadherin-1 (*Cdh1*), and cadherin-5 (*Cdh5*) were strongly downregulated (Fig. 1a).

When we examined the morphology of the BBB by transmission electron microscopy, cuprizone fed animals often displayed a discontinuous electron-dense junctional area with focally increased junctional width (Fig. 1c, Additional file 2: Figure S2), implying that the physical barrier of the BBB mediated by endothelial tight and adherens junctions could be altered. This observation was confirmed by immunofluorescence analysis of selected tight junction proteins that showed strongly reduced staining intensity in cuprizone fed animals (Fig. 1d–g). Increased BBB permeability was demonstrated by extravasation of tracers such as FITC-dextran (Fig. 1f, see also below). By electron microscopy we also observed locally split endothelial and astroglial basement membranes, hypertrophic astrocytes, and sporadic endothelial cells with atypical ultrastructure in cuprizone fed animals (Fig. 1c, Additional file 2: Figure S2). Moreover, while the number of blood vessels in the entire cross-sectional area of the corpus callosum remained unchanged, vessel density was strongly reduced (Fig. 1h), likely caused by the tissue swelling due to the dramatic gliosis at this time point (Additional file 2: Figure S1). Together, these data show that the increased BBB permeability at the peak of cuprizone induced demyelination coincides with substantial upregulation of BBB-disrupting pro-inflammatory mediators. This is associated with strong downregulation of endothelial tight junction proteins and morphological changes at the endothelial barrier.

### Cuprizone directly affects BBB permeability in vitro

We next explored whether cuprizone directly damages cellular constituents of the NVU, namely mouse brain endothelial cells or astrocytes, as observed for mature oligodendrocytes [10]. Using primary cultures of either cell type in a WST1 assay that measures the activity of cellular dehydrogenases, cuprizone did not affect metabolic activity within 24 h, even when administered in concentrations up to 500 μM (Additional file 2: Figure S3a, b). However, after 72 h incubation with 250 μM cuprizone, the WST1 signal in endothelial cells decreased by about 14% compared to vehicle treated cultures (Additional file 2: Figure S3c, d), which were not caused by cell death but rather reflected metabolic adaptations to the toxic cuprizone insult.

**Fig. 2:**
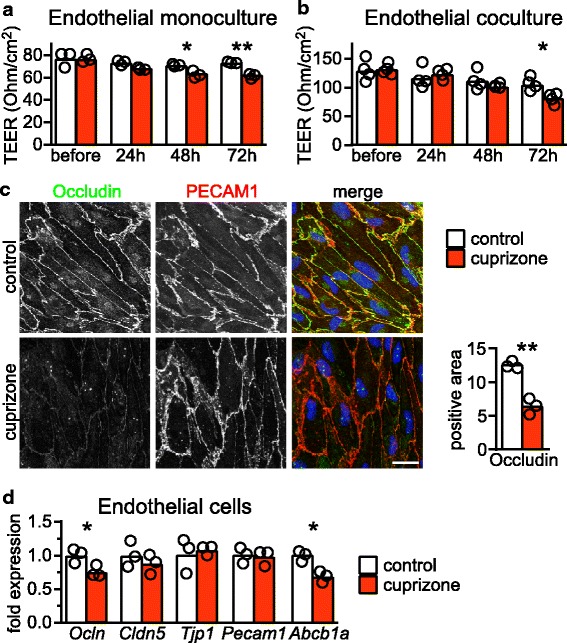
Cuprizone increases BBB permeability in vitro. Transendothelial electrical resistance (TEER) of (**a**) monocultures of primary endothelial cells (EC) and (**b**) co-cultures of EC with astrocytes after 250 μM cuprizone for 24 h, 48 h and 72 h. Evaluation of significance was done by 2way ANOVA with Sidak’s post test (*, *P* < 0.05; **, *P* < 0.01). Shown is one representative experiment but similar results were obtained in three additional experiments (each *N* = 3–4 mice per condition). **c** Immunostaining of EC for occludin and PECAM1 that had been treated 250 μM cuprizone for 48 h (Scale bar: 20 μm) with quantification of % anti-occludin positive area on the right (*N* = 3 independent experiments, Student’s t-test, *P* > 0.01 **). **d** Quantitative RT-PCR analysis on EC challenged with 250 μM cuprizone for 48 h. Results show mean fold changes of individual cultures (*N* = 3) normalized to vehicle treated controls (set to 1). Significance to control was evaluated by unpaired Student’s t-test (*P* < 0.05 *)

To investigate the effect of cuprizone on the barrier function of endothelial cells in more detail, we analyzed transendothelial electrical resistance (TEER) in an in vitro BBB system of endothelial monocultures. Surprisingly, cuprizone significantly decreased TEER after only 48 h that dropped further to about 80 ± 3% (mean ± SD) of control values after 72 h (Fig. 2a). This effect was also observed in the advanced BBB setup, in which endothelial cells were co-cultured with primary astrocytes (Fig. 2b). Moreover, by immunostaining and expression analysis of primary endothelial cells, we observed reduced protein and mRNA abundance of the tight junction protein occludin, and reduced *Abcb1a* mRNA of the ABCB1 (P-glycoprotein) efflux transporter, likely explaining the decreased tightness of the barrier in vitro (Fig. 2c, d). Abundance of mRNAs for the astroglial BBB maintenance factor sonic hedgehog and for other tight junction proteins, which were strongly downregulated in the corpus callosum of cuprizone treated animals (Fig. 1a, d, e), remained unchanged. Cuprizone treatment downregulated occludin in the absence of inflammatory mediators, whose expression in primary endothelial, astrocyte, and microglial cultures were not induced by the cuprizone challenge (Additional file 2: Figure S3e-g). Together, these in vitro data suggest that cuprizone could directly affect BBB constituents in vivo, even in the absence of additional disease processes.

### BBB dysfunction depends on local pathology

**Fig. 3:**
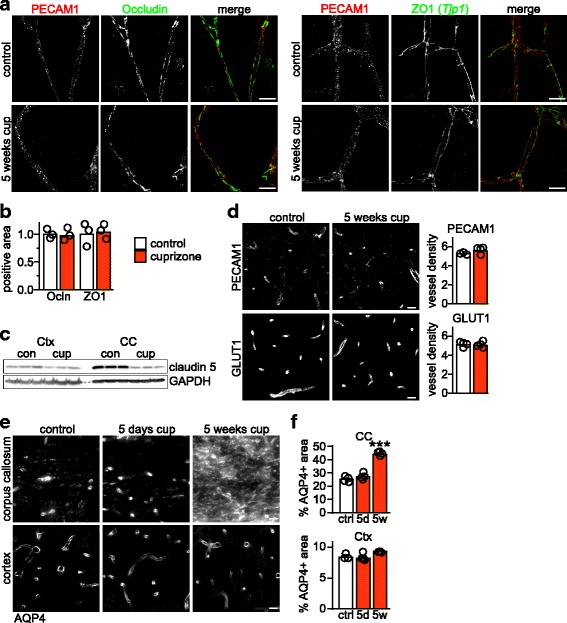
BBB dysfunction depends on local pathology. **a** Representative pictures of the cortex of untreated control mice and mice after 5 weeks cuprizone exposure immunostained for endothelial tight junction proteins as indicated co-labeled with the endothelial marker PECAM1 (Scale bars: 20 μm). **b** Quantification of abundance of the tight junction proteins occludin (Ocln) and ZO1 from pictures as in (**a**). **c** Immunoblot detecting claudin-5 in cortex (Ctx) and corpus callosum (CC) lysates from mice treated with cuprizone (cup) for 5 weeks and untreated controls. GAPDH shows equal loading of protein. **d** Immunostaining and density of blood vessels (PECAM1 or GLUT1 positive vessels per square mm) in the cortex of control mice (*N* = 5) and mice after 5 weeks cuprizone (*N* = 4). **e** Representative pictures of corpus callosum and cortex of untreated control mice and mice after 5 weeks cuprizone immunostained for AQP4 (Scale bar: 20 μm). **f** Quantification of immunolabeling as depicted in (**e**) of *N* = 3–4 animals per condition. Significance was evaluated by 1way ANOVA with Dunnett’s post test (***P < 0.001)

To test this, we treated mice for 5 weeks with cuprizone as before and then analyzed the integrity of endothelial tight junctions on cortical sections. The cortex develops the demyelinating pathology later than the corpus callosum (Additional file 2: Figure S1), as previously reported [13, 23], allowing us to test if tight junction integrity is affected before pathology exacerbates. In the cortex of cuprizone treated animals and controls, staining intensity and continuity of the tight junction proteins occludin and ZO1 was similar (Fig. 3a, b), in contrast to the strongly reduced staining intensity in corpus callosum of the same animals (Fig. 1d–g). Similarly, abundance of the tight junction protein claudin-5 (Fig. 3c) was not significantly reduced in cortex (90 ± 2% of controls in cortex lysates) of cuprizone fed mice in comparison to corpus callosum lysates (44 ± 4% of controls, *n* = 3, 2-way ANOVA *P* < 0.0001). Of note, abundance of claudin-5 in control animals was 69 ± 3% in cortex compared to corpus callosum (*n* = 3, *n* = 3, 2-way ANOVA *P* < 0.0001), in line with a study using porcine brain [41]. In accordance with the mild gliosis (Additional file 2: Figure S1), vessel density remained unchanged in cortex of cuprizone treated mice compared to controls (Fig. 3d). These findings suggest that in vivo, cuprizone alone is not sufficient to completely abolish BBB integrity (see also below).

To substantiate the heterogeneity of BBB pathology with another marker for NVU integrity, we compared Aquaporin 4 (AQP4) distribution in corpus callosum and cortex by immunostaining. AQP4 localization was restricted to astrocyte endfeet in control animals and the cortex of mice up to 5 weeks cuprizone (Fig. 3e, f), suggesting this brain region intact with respect to BBB integrity. In contrast, in the corpus callosum of these mice, AQP4 displayed a diffuse staining pattern, and *Aqp4* mRNA expression was significantly upregulated (2.71 ± 0.01 fold, *n* = 4 animals per group, *P* < 0.0001, Student’s t-test), a condition also found in MS lesions [50] and typical for cerebral edema in EAE. Together these findings suggest that the morphological disturbances at the BBB are associated with local active disease in cuprizone fed animals. This prompted us to investigate at which disease state BBB dysfunction develops.

### BBB impairment and edema are very early disease processes

**Fig. 4:**
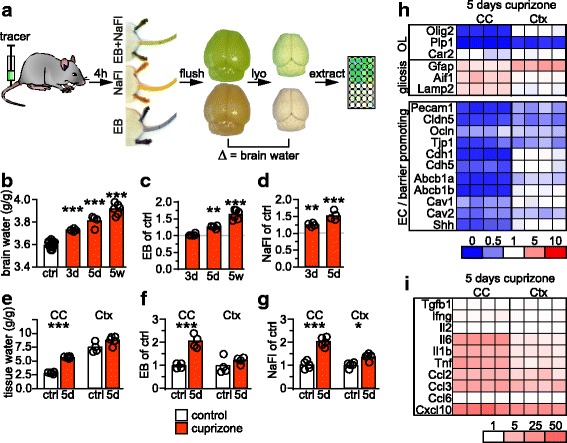
Cuprizone induces early BBB dysfunction and edema. **a** Experimental procedure to measure BBB permeability by quantifying extravasation of the tracers Evans blue (EB) and sodium fluorescein (NaFl), and edema (brain water). Tracers are i.v. injected, and mice are flushed with PBS after 4 h. The dissected brain is lyophilized (lyo). Brain water content (edema) is calculated from the weight difference of wet to lyophilized tissue. Tracers are extracted, and fluorescence intensity quantified. **b** Brain water (g/g dry brain) in mice fed cuprizone for 3 days to 5 weeks as indicated, and untreated controls (*N* = 4–5). Significance to control was evaluated by 1way ANOVA with Tukey’s post test (***P < 0.001). **c**, **d** Extravasated EB (*N* = 4–6) and NaFl (*N* = 4) after cuprizone administration for 3 days to 5 weeks. Significance to control was evaluated by 1way ANOVA with Tukey’s post test (**P < 0.01, ***P < 0.001). **e** Water content (g/g dry tissue) in dissected corpus callosum (CC) or cortex (Ctx) of mice fed cuprizone for 5 days or controls (*N* = 4–5). Significance was determined by unpaired Student’s t-test for each brain region (***P < 0.001). Extravasated (**f**) EB (*N* = 4) and (**g**) NaFl (*N* = 5) in dissected CC or Ctx of mice fed cuprizone for 5 days normalized to untreated controls. Significance to controls was evaluated by Student’s t-test (*P < 0.05, ***P < 0.001). Quantitative RT-PCR analysis on dissected CC or Ctx from mice that received cuprizone for 5 days (*N* = 4) or control mice, evaluating cellular markers (**h**) and inflammatory mediators (**i**). Values of individual mice are shown as fold differences to the mean of *N* = 4–5 controls (set to 1) according to color code

To determine the temporal progression of BBB impairment, we simultaneously assessed the degree of BBB permeability and vasogenic edema after 3 days to 5 weeks of cuprizone administration (Fig. 4a). Brain water as a measure of edema was already significantly evident after 3 days cuprizone and progressively increased (Fig. 4b). BBB dysfunction, as assessed by the biochemical extraction of extravasated Evans blue or sodium fluorescein (Fig. 4a), developed with a similar time course as edema, demonstrating a significant 1.25 ± 0.02 fold extravasation (mean ± s.e.m., *n* = 4) after 3 days of cuprizone (Fig. 4c, d). Because global edema values could be misleading, we determined water content individually, in corpus callosum and cortex of the same animals after 5 days of cuprizone exposure. While the water content in the cortex of cuprizone treated animals remained in the same range as in controls, the corpus callosum of cuprizone treated animals displayed a 16.6 ± 0.5% water increase (Fig. 4e, mean ± s.e.m., *N* = 4–5), a value typical for focal edema [28]. Corresponding with these local differences in edema, focal extravasation of fluorescent tracers increased more than twofold in the corpus callosum but only marginally in the cortex of cuprizone treated animals (Fig. 4f, g).

To assess how this regional heterogeneity of edema and BBB permeability correlates with local pathology and gliosis in this early disease phase, we performed a series of quantitative expression analyses on dissected corpus callosum and cortex together with histopathological evaluation of tissue sections, after 5 days of cuprizone. Expression of marker proteins for astrocytes and microglia reflected the mild histopathological changes (Fig. 4h, Additional file 1: Table S3, Additional file 2: Figure S1). Despite reduced *Plp1* and *Olig2* expression, mice lacked signs of demyelination and loss of oligodendrocytes, as reported previously [13, 24]. The abundance of mRNAs of endothelial tight junction proteins were only moderately downregulated in cortex compared to corpus callosum, mirroring the immunostaining data at 5 weeks of cuprizone exposure (Fig. 4h, compare Figs. 1d-e, 3a). Expression of the ABCB1 transporter and associated caveolins as well as of the BBB maintenance factor sonic hedgehog remained almost unaffected in the cortex compared to untreated wild type mice. It is possible that a direct effect of cuprizone contributed to the residual downregulation of endothelial markers and to the slight BBB hyperpermeability (Fig. 4g), similar to our observations in vitro (compare Fig. 2). In contrast, all markers for NVU integrity were strongly downregulated in corpus callosum at this early time point of cuprizone induced pathology.

Levels of the inflammatory mediators *Il6*, *Il1b*, *Tnf*, and *Ccl2* were strongly elevated in the corpus callosum but only moderately increased in the cortex after 5 days of cuprizone (Fig. 4i). These data demonstrate that the BBB integrity is already affected within the first days of cuprizone exposure, coinciding with elevated levels of inflammatory mediators but preceding overt demyelination and oligodendrocyte loss.

### Reduced inflammation ameliorates BBB pathology

These findings prompted us to test directly whether demyelination and oligodendrocyte loss or local gliosis and the secretion of inflammatory mediators correlate with BBB dysfunction. Therefore, we used type 3 CXC chemokine receptor (CXCR3) deficient mice [26] that develop demyelination in response to cuprizone as wild type mice but show strongly reduced reactive gliosis and expression of pro-inflammatory cytokines and chemokines such as TNF, IL6 and CCL2 [32].

**Fig. 5:**
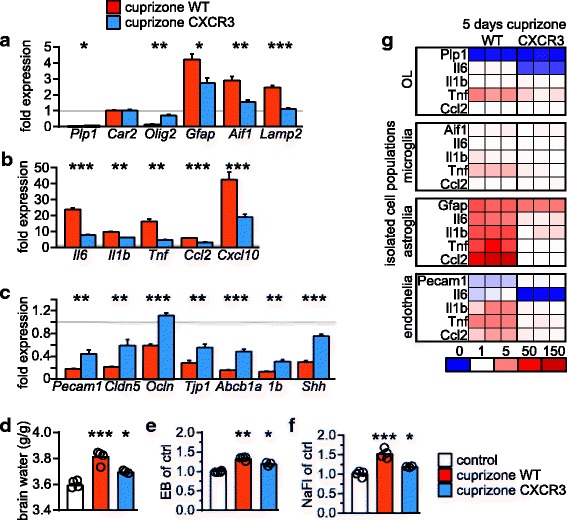
CXCR3 KO ameliorates BBB pathology. **a**-**c** Quantitative RT-PCR analysis on dissected corpus callosum tissue samples from wild type (red) or CXCR3 deficient (blue) mice after 5 days cuprizone administration or untreated control mice. Results are expressed as mean fold change of *N* = 3–4 mice ± s.e.m., normalized to untreated wild type control animals (set to 1; gray line, *N* = 5). Assessed were (**a**) cellular markers, (**b**) cytokines and chemokines, and (**c**) endothelial and BBB promoting markers as indicated. Significance between WT mice and CXCR3 mice was evaluated by Student’s t-test for each individual gene (*P < 0.05, **P < 0.01, ***P < 0.001). **d**-**f** Brain water (d, g/g dry brain), of extravasated EB (**e**), and NaFl (**f**) in untreated wild type controls (white, *N* = 3–4), and wild type (red, *N* = 3–4) or CXCR3 deficient (blue, *N* = 3) mice exposed to cuprizone for 5 days. Significance to control was evaluated by 1way ANOVA with Tukey’s post test (*P < 0.05, **P < 0.01, ***P < 0.001). **g** Quantitative RT-PCR on isolated cells as indicated from brain of from WT or CXCR3 deficient mice after 5 days cuprizone administration or untreated control mice. Results show fold-change values according to color code of individual animals (*N* = 3) normalized to the mean of untreated control animals (set to 1; *N* = 5)

After 5 days of cuprizone, we found attenuated expression of markers for astrogliosis and microgliosis as well as inflammatory mediators in CXCR3 deficient corpus callosum compared to identically treated wild type animals (Fig. 5a, b). Expression of the oligodendroglial transcription factor Olig2 and the myelin protein PLP1 was ameliorated in CXCR3 deficient mice (*Olig2*, 3.86 ± 0.23 fold; *Plp1*, 2.28 ± 0.05 fold in CXCR3 knockout mice compared to cuprizone fed controls), suggesting that the oligodendroglial damage was slightly less severe at this time point. Interestingly, the strong downregulation of genes indicative of BBB dysfunction such as tight junction proteins and BBB maintenance factors was also ameliorated in CXCR3 deficient animals (Fig. 5c). Reduced brain edema (Fig. 5d) and attenuated extravasation of fluorescent tracers (Fig. 5e, f) in CXCR3 deficient animals further support the hypothesis that pro-inflammatory mediators contribute to BBB disruption in response to cuprizone exposure.

Although CXCR3 is mainly expressed by microglial cells in untreated mice [26], and also when mice are challenged with cuprizone (Additional file 2: Figure S4), the cell type responsible for establishing the cytokine milieu during initial cuprizone pathology that contributes to BBB dysfunction is unknown. Therefore, we acutely isolated microglia, astrocytes, oligodendrocytes, and endothelial cells from wild type and CXCR3 deficient mice after 5 days of cuprizone treatment and from untreated wild type control animals, and analyzed mRNA abundance of *Tnf*, *Il1b*, *Il6*, and *Ccl2*. We chose these inflammatory mediators because their expression pattern correlates with the extent of BBB disturbances: after 5 days of cuprizone, their expression levels were most strongly increased in corpus callosum of wild type mice, moderately increased in the corpus callosum of CXCR3 mutant animals, and only weakly upregulated in the cortex of wild type animals compared to untreated wild type controls (compare Figs. 4i and 5b). Oligodendroglia did not significantly contribute to the cytokine and chemokine profile after 5 days of cuprizone and surprisingly, neither did microglia (Fig. 5g, Additional file 1: Table S4). Endothelial cells showed moderate upregulation of cytokine and chemokine expression. In contrast, we identified astrocytes as the major source of all tested pro-inflammatory mediators at this early disease phase (Fig. 5g). Further, the increased expression of inflammatory molecules was completely abolished in astroglia of CXCR3 deficient mice, suggesting that microglial CXCR3 signaling induces astroglial upregulation of cytokines and chemokines in response to cuprizone.

## Discussion

BBB impairment is considered as an important feature in MS pathologies, but the causal relation to disease processes during initial lesion formation and its impact on disease activity is unknown. In the current study we analyzed the temporal and spatial relationship of BBB dysfunction to gliosis, expression of inflammatory mediators, and demyelinating pathology in the cuprizone mouse model of demyelination. By using wild type and CXCR3 deficient mice, we demonstrate that BBB impairment is most pronounced in the corpus callosum of wild type animals, to a lesser extent in the corpus callosum of CXCR3 deficient mice, and only minimally in the cortex of wild type mice in the initial disease phase before the onset of demyelination. Our data indicate that IL6, IL1β, TNF, and CCL2 are the most likely candidates to contribute to BBB dysfunction in the cuprizone model.

It is well known that TNF, IL6, IL1β and CCL2 can each induce downregulation of endothelial tight junction proteins in vitro and induce BBB hyperpermeability in vivo [14, 20, 43, 47, 53, 58]. Enhanced paracellular leakage is mediated by downregulation of mRNA of tight junction proteins as shown in our study and in a model of bacterial infection [40]. Pro-inflammatory cytokines induce signaling cascades that can lead to downregulation of stabilizing factors such as sonic hedgehog [3, 58], to activation of effector proteins such as matrix metalloproteinases [33], and to the formation of reactive oxygen species [47]. In our experimental paradigm, increased expression of nitric oxide synthases and downregulated sonic hedgehog signaling likely contributed to tight junction disruption. In addition, all three cytokines have the ability to reduce ABCB1 mediated efflux, facilitating transendothelial passage [25]. We found downregulation of ABCB1 not only in cuprizone fed mice but also in endothelial cultures in the absence of inflammatory mediators, suggesting that cuprizone directly affected transendothelial passage.

Although it is generally assumed that microglia secrete the majority of effector molecules, our data show that in the initial disease phase of the cuprizone model microglia do not themselves contribute to the upregulation of IL6, IL1β, TNF or CCL2; rather astrocytes (with a moderate participation by endothelial cells) are the main source of these pro-inflammatory cytokines and chemokines. At later disease stages, however, microglia substantially add to the production of inflammatory factors [57]. Astrocytes participate in recruiting microglia as shown in mouse mutants with acutely depleted astrocytes [52]. They promote recovery and repair in mouse models of remyelination but can also facilitate demyelination in acute active lesions of MS patients [11, 31, 35]. Whether astrocytes are the main source of disease promoting factors in presymptomatic MS patients before the onset of demyelination is unknown. We demonstrate that astrocytes, which are intimately involved in regulating BBB function via their endfeet, create a local inflammatory milieu that likely participates in destabilizing BBB integrity. Although cuprizone by itself does not induce astroglial, endothelial, or microglial expression of any of the tested pro-inflammatory mediators in vitro, it mildly affects metabolic activity in endothelial cells (this study) in addition to mature oligodendrocytes [10]. Death of oligodendrocytes in vivo is enforced by local glial activation [27]. We hypothesize that also vascular cells contribute to this complex crosstalk that lead to BBB impairment and demyelination in the cuprizone model.

Paracellular influx of fluid because of BBB disruption is the leading cause of vasogenic edema [54], also found in MS and inflammatory models of MS [9, 48, 60]. Edema correlates with increased AQP4 abundance and its mislocalization from (frequently hypertrophic) astroglial endfeet that is associated with altered basement membrane morphology in EAE [2, 60]. We also observed loosening of the astrocytic and endothelial basement membranes in the cuprizone model, emphasizing similar pathogenic processes in these disparate models of MS. Although it is well-known that cuprizone intoxication causes spongiform degeneration of the CNS [15], edema has been largely ignored, potentially because of the absence of massive BBB disruption in this model [8, 12, 30]. For the first time, to our knowledge, we quantified edema in the cuprizone model and found that increased brain water content was most pronounced during overt demyelination. Importantly, edema was already obvious before the onset of demyelination and oligodendrocyte loss.

In agreement with others [13, 23], we observed marked regional differences in disease manifestation in the cuprizone model. Interestingly, similar to our observation in the cortex of cuprizone treated animals, cortical pathology in MS also differs substantially from white matter lesions; the former comprising only mild gliosis and modest alteration of tight junctions [21, 56]. In addition to the differences in tissue architecture and expression profiles of neural cells [22, 38], the regional heterogeneity of pathology could also be influenced by differences in vasculature. As we show here in accordance with previous studies, vessel density in gray matter is about double of that in white matter, and steady state levels of tight junction proteins are lower [41]. Together, these differences likely modify BBB properties, which might play a role in rendering cortical MS lesions less susceptible to disease exacerbation.

Does an impaired BBB directly affect the course of demyelinating disease? Chronic upregulation of inflammatory mediators directly impairs BBB integrity and can induce demyelinating pathology [46]; conversely, their genetic or pharmacological reduction can improve BBB function but their role in modulating severity of demyelinating disease in the context of EAE or cuprizone is less clear [7, 16, 36, 39]. The induction of an inflammatory milieu and BBB impairment have been uncoupled by inhibition of nitric oxide synthesis in the cuprizone model [29] or by targeted overexpression of claudin-1 in EAE [44] that both support BBB tightness but presumably do not (directly) affect expression of pro-inflammatory molecules. In these experimental paradigms, clinical symptoms of treated/transgenic mice were ameliorated, suggesting that endothelial integrity might contribute to disease expression. In a study with 39 patients with neuromyelitis optica, BBB leakage in normal appearing white matter correlated with progression to MS pathology [17]. We show here that BBB dysfunction and edema occurred before demyelination, suggesting that demyelination itself does not cause BBB damage. We speculate that BBB dysfunction might serve as a predictive marker for local disease activity.

## Conclusions

In summary, our data show that in vitro cuprizone directly increases BBB permeability mediated by downregulation of tight junction proteins. In vivo, astroglial derived pro-inflammatory cytokines create a local inflammatory milieu that likely further destabilizes local BBB integrity and induces edema. We envision that glial activation and production of pro-inflammatory mediators in any neurological disorder destabilizes BBB integrity. In demyelinating disease, these presymptomatic disease processes might have prospective value for future disease activity and disease progression.

## Additional files

Additional file 1: **Table S1.** Primer sequences used for gene expression analyses. **Table S2.** Quantification of gene expression in corpus callosum. **Table S3.** Quantification of gene expression in cortex. **Table S4.** Quantification of gene expression in acutely isolated cells. (PDF 70 kb)

Additional file 2: **Figure S1.** Time course of cuprizone induced pathology in corpus callosum and cortex. (a) Representative pictures of the corpus callosum (CC) and the cortex (Ctx) of untreated control mice and mice after cuprizone exposure for 5 days and 5 weeks assessing myelination (Gallyas silver impregnation), mature oligodendrocytes (CAII), activated microglia (MAC3), and astrocytes (GFAP) (Scale bars: 20 μm) with quantification in (b). Each bar represents the mean value of *N* = 3–4 animals per condition with individual data points. Significance to control was evaluated by 1way ANOVA with Tukey’s post test (*P < 0.05, **P < 0.01, ***P < 0.001). **Figure S2.** Endothelial junctions in mice after 5 weeks cuprizone. Electron microscopic images of capillaries showing disconnected endothelial and astroglial basement membranes (a, arrowheads), an affected endothelial cell (b) with electron light cytoplasm and focal disruption of endothelial tight junctions (a, b, arrow). Astroglial endfeet often appeared swollen (c, green). Scale bars: 2 μm (AC, astrocyte; EC, endothelial cell; EF, astroglial endfoot; M, microglia/macrophage; P, pericyte). **Figure S3.** Cuprizone affects endothelial cells but not astrocytes in vitro*.* Cell vitality measurements (WST1 assays) of primary (a, c) endothelial cells or (b, d) astrocytes after exposure to vehicle (0, white) or to increasing concentrations of cuprizone (50–500 μM, red) for 24 h (a, b) or with 250 μM cuprizone for 72 h (c, d) (*N* = 5 per condition). Incubation with 20 μM peroxide for 4 h induced cell death and was used as positive control (*N* = 3, +). Significance to vehicle was evaluated by (a, b) 1way ANOVA with Dunnett’s post test or (c, d) Student’s t-test (**P < 0.01, ***P < 0.001). (e, f, g) Quantitative RT-PCR analysis on cultured primary endothelial cells (e), astrocytes (f), and microglia (g) challenged with 250 μM cuprizone for 48 h. Results show mean fold change with individual data points of *N* = 3 cultures normalized to vehicle control (set to 1). **Figure S4.** CXCR3 is expressed by microglia in cuprizone fed mice. Direct GFP fluorescence of CXCR3^GFP/GFP^ mice together with immunolabeling of cell type specific markers for microglia (Iba1), astrocytes (GFAP), or oligodendroglia (Olig2) in the corpus callosum and cortex in CXCR3 deficient mice that had been exposed to cuprizone for 5 days (Scale bars: 20 μm). (PDF 19968 kb)
