# Supplementary material for: Blood-brain barrier hyperpermeability precedes demyelination in the cuprizone model
Source: Acta Neuropathol Commun. 2017 Dec 1;5:94. doi: 10.1186/s40478-017-0497-6 (PMC5710130; doi:10.1186/s40478-017-0497-6)
Supplement: Supplementary file 1 — Table S1. Primer sequences used for gene expression analyses. Table S2. Quantification of gene expression in corpus callosum. Table S3. Quantification of gene expression in cortex. Table S4. Quantification of gene expression in acutely isolated cells. (PDF 70 kb) [file 40478_2017_497_MOESM1_ESM.pdf]

Table S1. Primer sequences used for gene expression analyses.
All primers used for expression analysis are intron-spanning.

**Mouse**	**Primer sequence 5'-3'; forward – reverse**
Rplp0	GATGCCCAGGGAAGACAG – ACAATGAAGCATTTTGGATAATCA
Hprt	TCCTCCTCAGACCGCTTTT – CCTGGTTCATCATCGCTAATC
Plp1	TCAGTCTATTGCCTTCCCTAGC – AGCATTCCATGGGAGAACAC
Car2	CAAGCACAACGGACCAGA – ATGAGCAGAGGCTGTAGG
Olig2	AGACCGAGCCAACACCAG – AAGCTCTCGAATGATCCTTCTTT
Aif1	TGTTTTTCTCCTCATACATCAGAATC – CCGAGGAGACGTTCAGCTAC
Lamp2	AAGGTGCAACCTTTTAATGTGAC – TGTCATCATCCAGCGAACAC
Gfap	TGCTCCTGCTTCGAGTCCTT – CAAGAGGAACATCGTGGTAAAGA
Ocln	TCCGTGAGGCCTTTTGAA – GGTGCATAATGATTGGGTTTG
Aqp4	TGGAGGATTGGGAGTCACC – TGAACACCAACTGGAAAGTGA
Cldn5	ACGGGAGGAGCGCTTTAC – GTTGGCGAACCAGCAGAG
Tjp1	ATGCAGACCCAGCAAAGGT – TGACCAAGAGCTGGTTGTTTT
Cdh1	ATCCTCGCCCTGCTGATT – ACCACCGTTCTCCTCCGTA
Cdh5	TCATCAAACCCACGAAGTCC – GGTCTGTGGCCTCAATGTAGA
Pecam1	GCTGGTGCTCTATGCAAGC – ATGGATGCTGTTGATGGTGA
Shh	TCCACTGTTCTGTGAAAGCAG – GGGACGTAAGTCCTTCACCA
Cav1	ACCCCAAGCATCTCAACG – CACAGTGAAGGTGGTGAAGC
Cav2	CCTCACCAGCTCAACTCTCA – TCACATATTTGCTGATTTCAAAGAG
Abcb1a	GGGCATTTACTTCAAACTTGTCA – TTTACAAGCTTCATTTCCTAATTCAA
Abcb1b	AGTGGACCCAACAGTACTCTGAT – GCACCAATCCCGGTGTAATA
Vegfa	TTACTGCTGTACCTCCACC – ACAGGACGGCTTGAAGATG
Hif1a	CATGATGGCTCCCTTTTTCA – GTCACCTGGTTGCTGCAATA
Nos2	TGAACTTGAGCGAGGAGCA – TTCATGATAACGTTTCTGGCTCT
Nos3	CCAGTGCCCTGCTTCATC – GCAGGGCAAGTTAGGATCAG
Tgfb1	TGGAGCAACATGTGGAACTC – CAGCAGCCGGTTACCAAG
Mmp9	CAGAGGTAACCCACGTCAGC – GGGATCCACCTTCTGAGACTT
Tnf	TGCCTATGTCTCAGCCTCTTC – GAGGCCATTTGGGAACTTCT
Ifng	TCAAGTGGCATAGATGTGGAAGAA – TGGCTCTGCAGGATTTTCATG
Il1b	GCTACCAAACTGGATATAATCAGGA – CCAGGTAGCTATGGTACTCCAGAA
Il2	CGCAGAGGTCCAAGTTCATCT – CGCAGAGGTCCAAGTTCATCT
Il6	GCTACCAAACTGGATATAATCAGGA – CCAGGTAGCTATGGTACTCCAGAA
Ccl2	GCCTGCTGTTCACAGTTGC – CAGGTGAGTGGGGCGTTA
Ccl3	TGCCCTTGCTGTTCTTCTCT – GTGGAATCTTCCGGCTGTAG
Ccl6	TCTTTATCCTTGTGGCTGTCC – TGGAGGGTTATAGCGACGAT
Cxcl10	GCTGCCGTCATTTTCTGC – TCTCACTGGCCCGTCATC

**Rat**	
Rplp0	GATGCCCAGGGAAGACAG – ACAATGAAGCATTTTGGATAATCA
Hprt	GGTCCATTCCTATGACTGTAGATTTT – CAATCAAGACGTTCTTTCCAGTT
Ocln	ATCTAGAGCCTGGAGCAACG – GTCAAGGCTCCCAAGACAAG
Cldn5	GGTCTTTAGCCATGGGGTCT – CAGCCTACCAGACACAGCAC
Tjp1	GCATGTAGACCCAGCAAAGG – GGTTTTGTCTCATCATTTCCTCA
Pecam1	CTCAGTCGGCTGACAAGATG – AGGCTTGCATAGAGCAGCAT
Abcb1a	GAAAGGAATTTACTTCAAACTTGTCA – CACAAGCTTCATTTCCTAATTCAA
Tgfb1	GGACTCTCCACCTGCAAGAC – GACTGGCGAGCCTTAGTTTG
Tnf	TGTCTGTGCCTCAGCCTCTTC – GAGCCCATTTGGGAACTTCT
Il6	CCCTTCAGGAACAGCTATGAA – ACAACATCAGTCCCAAGAAGG
Ccl2	CAGAAACCAGCCAACTCTCA – GTGGGGCATTAACTGCATCT

Table S2. Quantification of gene expression in corpus callosum.

Wild type animals were treated with cuprizone for 5 days or 5 weeks or left untreated, and gene expression was determined in dissected corpus callosum. Data are expressed as mean of N=4-5 animals ± SEM normalized to untreated controls. Significance (P value) was determined by Student's t-test (n.d., not determined).

	**Fold expression**		**Significance to control (P)**	
**Gene symbol**	**5 days cuprizone**	**5 weeks cuprizone**	**5 days cuprizone**	**5 weeks cuprizone**
Gfap	4.26 ± 0.29	11.16 ± 0.78	0.0000	0.0000
Aqp4	1.28 ± 0.10	2.74 ± 0.01	0.1520	0.0001
Aif1	2.91 ± 0.25	7.55 ± 0.68	0.0000	0.0000
Lamp2	2.49 ± 0.09	3.25 ± 0.21	0.0001	0.0000
Plp1	0.04 ± 0.01	0.28 ± 0.07	0.0000	0.0024
Car2	1.02 ± 0.02	0.15 ± 0.03	0.8941	0.0002
Olig2	0.15 ± 0.01	0.39 ± 0.06	0.0000	0.0043
Pecam1	0.18 ± 0.02	0.16 ± 0.01	0.0000	0.0000
Ocln	0.62 ± 0.01	0.55 ± 0.02	0.0019	0.0000
Cldn5	0.22 ± 0.01	0.33 ± 0.03	0.0003	0.0122
Tjp1	0.29 ± 0.05	0.27 ± 0.02	0.0008	0.0016
Cdh1	0.13 ± 0.01	0.88 ± 0.02	0.0032	0.9066
Cdh5	0.33 ± 0.02	0.50 ± 0.04	0.0000	0.0001
Shh	0.48 ± 0.09	0.14 ± 0.03	0.0379	0.0005
Vegfa	0.05 ± 0.01	0.08 ± 0.01	1.0000	0.0316
Hif1a	0.23 ± 0.03	0.55 ± 0.05	0.0732	0.0000
Abcb1a	0.17 ± 0.01	n.d.	0.0000	-
Abcb1b	0.14 ± 0.01	n.d.	0.0000	-
Cav1	0.59 ± 0.04	3.16 ± 0.16	0.0031	0.0000
Cav2	0.48 ± 0.04	0.69 ± 0.04	0.0002	0.0018
Nos2	1.48 ± 0.02	5.86 ± 0.33	0.0000	0.0002
Nos3	1.70 ± 0.03	4.07 ± 0.23	0.0002	0.0000
Tgf1b	1.13 ± 0.04	5.99 ± 0.60	0.1387	0.0000
Tnf	16.08 ± 1.85	58.85 ± 2.90	0.0000	0.0000
Ifng	1.58 ± 0.10	1.86 ± 0.20	0.0354	0.0272
Il1b	9.70 ± 0.67	58.52 ± 5.10	0.0000	0.0000
Il2	1.01 ± 0.01	1.37 ± 0.06	1.0000	0.0369
Il6	23.65 ± 1.01	9.43 ± 1.37	0.0000	0.0001
Ccl2	5.89 ± 0.40	273.20 ± 21.5	0.0000	0.0000
Ccl3	14.60 ± 1.41	231.15 ± 25.0	0.0000	0.0000
Ccl6	1.19 ± 0.05	144.95 ± 12.9	0.0629	0.0000
Cxcl10	42.64 ± 4.86	62.67 ± 4.15	0.0000	0.0000

Table S3. Quantification of gene expression in cortex.
Wild type animals were treated with cuprizone for 5 days or left untreated, and gene expression was determined in dissected cortex. Data are expressed as mean of N=4 animals ± SEM normalized to untreated controls. Significance (P value) was determined by Student's t-test.

Gene symbol	Fold expression	Significance to control (P)
Gfap	4.24 ± 0.17	0.0000
Aqp4	0.99 ± 0.02	0.8514
Aif1	1.29 ± 0.03	0.0110
Lamp2	0.99 ± 0.04	0.8964
Plp1	0.17 ± 0.01	0.0000
Car2	1.06 ± 0.04	0.4867
Olig2	1.05 ± 0.03	0.6113
Pecam1	0.64 ± 0.05	0.0254
Ocln	0.76 ± 0.02	0.0034
Cldn5	0.49 ± 0.04	0.0009
Tjp1	0.58 ± 0.05	0.0060
Cdh1	1.09 ± 0.06	0.4855
Cdh5	0.99 ± 0.04	0.7884
Shh	1.06 ± 0.07	0.7815
Vegfa	1.07 ± 0.07	0.5894
Abcb1a	0.77 ± 0.03	0.0045
Abcb1b	1.01 ± 0.04	0.9245
Cav1	1.07 ± 0.05	0.5052
Cav2	0.76 ± 0.00	0.0298
Nos2	1.09 ± 0.01	0.0996
Nos3	1.08 ± 0.03	0.2227
Tgf1b	0.99 ± 0.11	0.8205
Tnf	2.36 ± 0.17	0.0005
Ifng	1.17 ± 0.10	0.4034
Il1b	2.30 ± 0.12	0.0017
Il2	1.03 ± 0.04	0.8974
Il6	2.12 ± 0.11	0.0010
Ccl2	3.43 ± 0.37	0.0014
Ccl3	5.44 ± 0.66	0.0001
Ccl6	1.06 ± 0.03	0.2905
Cxcl10	25.23 ± 1.12	0.0000

Table S4. Quantification of gene expression in acutely isolated cells.
Wild type or CXCR3 deficient animals were treated with cuprizone for 5 or left untreated, and cells were isolated by magnetic bead assisted sorting followed by gene expression in samples of individual mice. Data are expressed as mean of N=3-4 animals ± SEM normalized to untreated controls. Significance (P value) was determined by Student's t-test.

**Gene symbol**	**Fold expression**		
**Oligodendroglia**	**Wild type**	**CXCR3**	**Significance (P)**
Plp	0.05 ± 0.01	0.05 ± 0.01	0.7238
Tnf	4.46 ± 0.25	2.00 ± 0.26	0.0036
Il1b	1.09 ± 0.03	1.21 ± 0.05	0.1515
Il6	1.08 ± 0.03	0.26 ± 0.01	0.0000
Ccl2	1.04 ± 0.02	1.08 ± 0.01	0.2341
**Microglia**	**Wild type**	**CXCR3**	**Significance (P)**
Aif1	1.38 ± 0.08	1.26 ± 0.08	0.4431
Tnf	3.13 ± 0.08	1.31 ± 0.10	0.0011
Il1b	1.47 ± 0.15	1.10 ± 0.03	0.0954
Il6	1.09 ± 0.02	1.08 ± 0.01	0.5409
Ccl2	1.09 ± 0.04	1.18 ± 0.04	0.2709
**Astroglia**	**Wild type**	**CXCR3**	**Significance (P)**
Gfap	18.37 ± 1.87	8.64 ± 0.52	0.0061
Tnf	43.74 ± 4.51	1.36 ± 0.11	0.0000
Il1b	24.49 ± 0.63	1.84 ± 0.31	0.0004
Il6	11.55 ± 2.19	1.77 ± 0.13	0.0019
Ccl2	116.96 ± 16.45	1.08 ± 0.09	0.0000
**Endothelial cells**	**Wild type**	**CXCR3**	**Significance (P)**
Pecam1	0.74 ± 0.01	1.04 ± 0.04	0.0099
Tnf	6.48 ± 0.31	1.42 ± 0.10	0.0001
Il1b	4.14 ± 0.71	1.16 ± 0.17	0.0131
Il6	0.93 ± 0.06	0.02 ± 0.01	0.0001
Ccl2	3.50 ± 0.39	1.40 ± 0.07	0.0049
