# Supplementary material for: Blood-brain barrier hyperpermeability precedes demyelination in the cuprizone model
Source: Acta Neuropathol Commun. 2017 Dec 1;5:94. doi: 10.1186/s40478-017-0497-6 (PMC5710130; doi:10.1186/s40478-017-0497-6)
Supplement: Supplementary file 2 — Figure S1. Time course of cuprizone induced pathology in corpus callosum and cortex. (a) Representative pictures of the corpus callosum (CC) and the cortex (Ctx) of untreated control mice and mice after cuprizone exposure for 5 days and 5 weeks assessing myelination (Gallyas silver impregnation), mature oligodendrocytes (CAII), activated microglia (MAC3), and astrocytes (GFAP) (Scale bars: 20 μm) with quantification in (b). Each bar represents the mean value of N = 3–4 animals per condition with individual data points. Significance to control was evaluated by 1way ANOVA with Tukey’s post test (*P < 0.05, **P < 0.01, ***P < 0.001). Figure S2. Endothelial junctions in mice after 5 weeks cuprizone. Electron microscopic images of capillaries showing disconnected endothelial and astroglial basement membranes (a, arrowheads), an affected endothelial cell (b) with electron light cytoplasm and focal disruption of endothelial tight junctions (a, b, arrow). Astroglial endfeet often appeared swollen (c, green). Scale bars: 2 μm (AC, astrocyte; EC, endothelial cell; EF, astroglial endfoot; M, microglia/macrophage; P, pericyte). Figure S3. Cuprizone affects endothelial cells but not astrocytes in vitro. Cell vitality measurements (WST1 assays) of primary (a, c) endothelial cells or (b, d) astrocytes after exposure to vehicle (0, white) or to increasing concentrations of cuprizone (50–500 μM, red) for 24 h (a, b) or with 250 μM cuprizone for 72 h (c, d) (N = 5 per condition). Incubation with 20 μM peroxide for 4 h induced cell death and was used as positive control (N = 3, +). Significance to vehicle was evaluated by (a, b) 1way ANOVA with Dunnett’s post test or (c, d) Student’s t-test (**P < 0.01, ***P < 0.001). (e, f, g) Quantitative RT-PCR analysis on cultured primary endothelial cells (e), astrocytes (f), and microglia (g) challenged with 250 μM cuprizone for 48 h. Results show mean fold change with individual data points of N = 3 cultures normalized to vehicle control (set t [file 40478_2017_497_MOESM2_ESM.pdf]

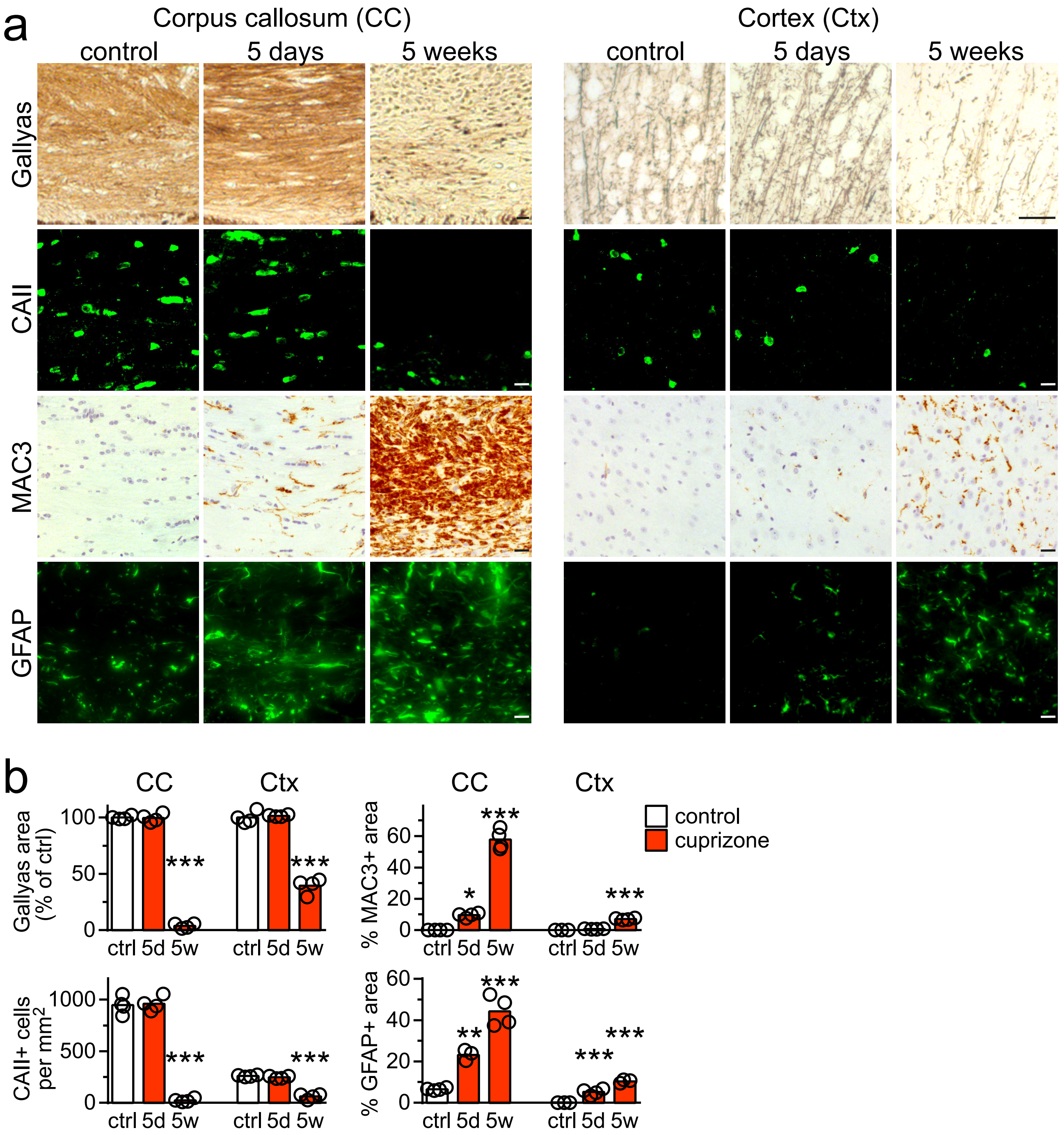

Figure S1. Time course of cuprizone induced pathology in corpus callosum and cortex.
(a) Representative pictures of the corpus callosum (CC) and the cortex (Ctx) of untreated control mice and mice after cuprizone exposure for 5 days and 5 weeks assessing myelination (Gallyas silver impregnation), mature oligodendrocytes (CAII), activated microglia (MAC3), and astrocytes (GFAP) (Scale bars: 20 μm) with quantification in (b). Each bar represents the mean value of N=3-4 animals per condition with individual data points. Significance to control was evaluated by 1way ANOVA with Tukey's post test (*P < 0.05, **P < 0.01, ***P < 0.001).

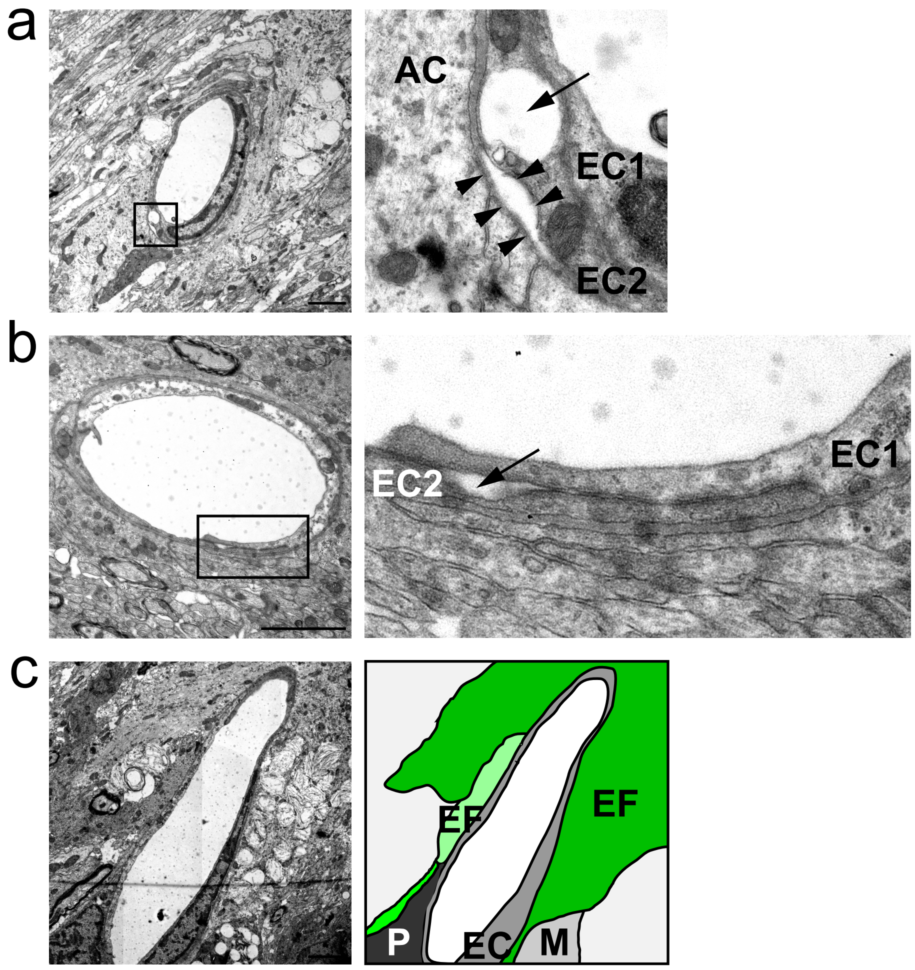

Figure S2. Endthelial junctions in mice after 5 weeks cuprizone.
Electron microscopic images of capillaries showing disconnected endothelial and astroglial basement membranes (a, arrowheads), an affected endothelial cell (b) with electron light cytoplasm and focal disruption of endothelial tight junctions (a, b, arrow). Astroglial endfeet often appeared swollen (c, green). Scale bars: 2 µm (AC, astrocyte; EC, endothelial cell; EF, astroglial endfoot; M, microglia/macrophage; P, pericyte).

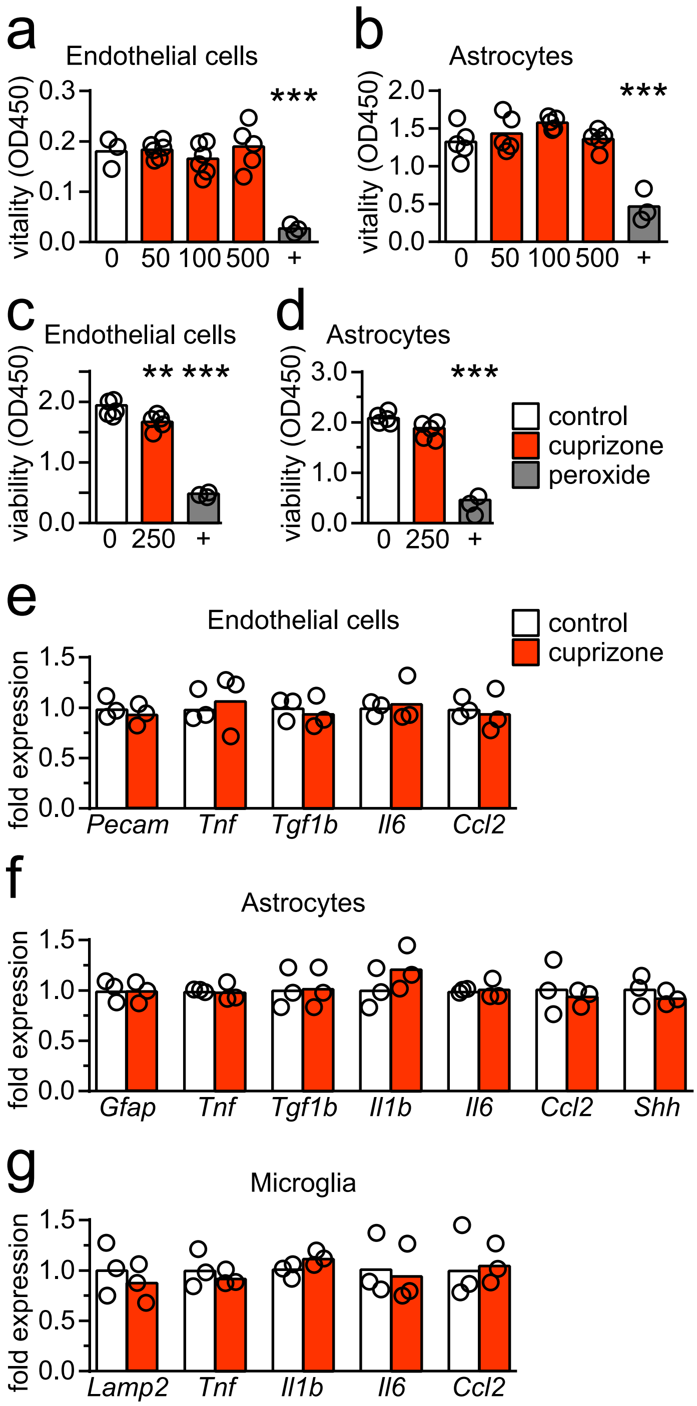

Figure S3: Cuprizone affects endothelial cells but not astrocytes *in vitro*. Cell vitality measurements (WST1 assays) of primary (a, c) endothelial cells or (b, d) astrocytes after exposure to vehicle (0, white) or to increasing concentrations of cuprizone (50-500 µM, red) for 24h (a, b) or with 250 µM cuprizone for 72h (c, d) (N=5 per condition). Incubation with 20 µM peroxide for 4h induced cell death and was used as positive control (N=3, +). Significance to vehicle was evaluated by (a, b) 1way ANOVA with Dunnett's post test or (c, d) Student's t-test (**P<0.01, ***P<0.001). (e, f, g) Quantitative RT-PCR analysis on cultured primary endothelial cells (e), astrocytes (f), and microglia (g) challenged with 250 µM cuprizone for 48h. Results show mean fold change with individual data points of N=3 cultures normalized to vehicle control (set to 1).

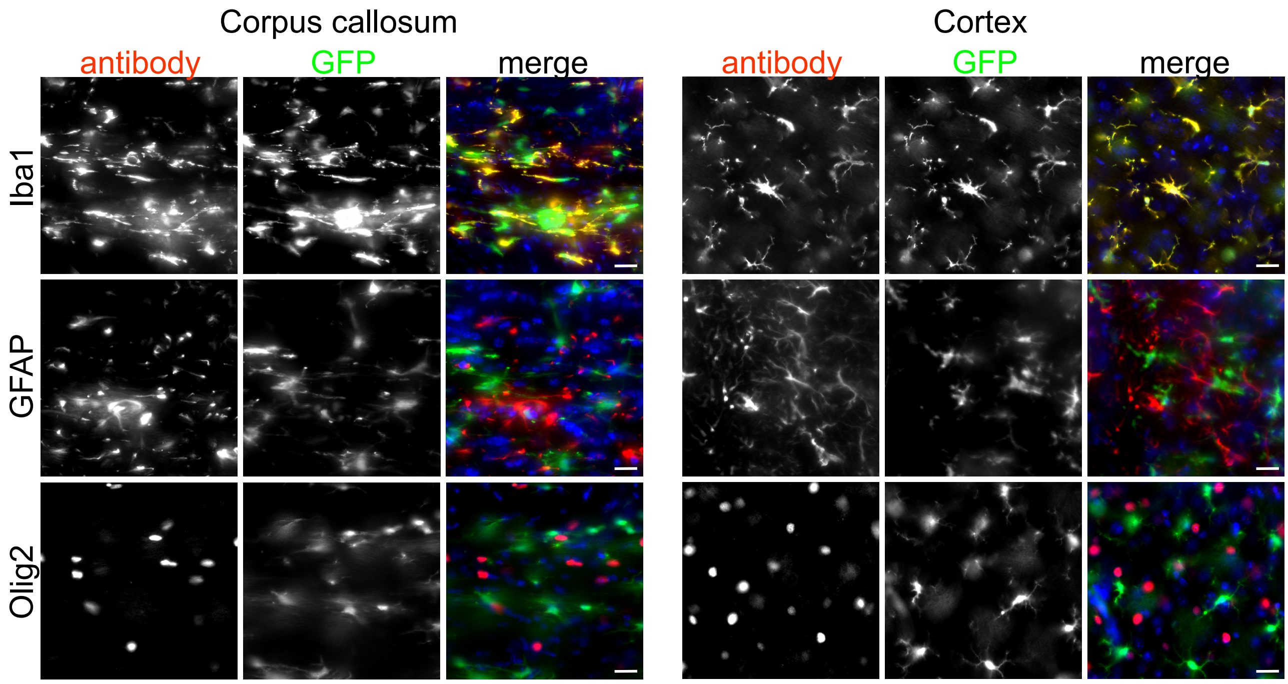

Figure S4. CXCR3 is expressed by microglia in cuprizone fed mice.
Direct GFP fluorescence of CXCR3$^{GFP/GFP}$ mice together with immunolabeling of cell type specific markers for microglia (Iba1), astrocytes (GFAP), or oligodendroglia (Olig2) in the corpus callosum and cortex in CXCR3 deficient mice that had been exposed to cuprizone for five days (Scale bars: 20 µm).
